# The lichen secondary metabolite atranorin suppresses lung cancer cell motility and tumorigenesis

**DOI:** 10.1038/s41598-017-08225-1

**Published:** 2017-08-15

**Authors:** Rui Zhou, Yi Yang, So-Yeon Park, Thanh Thi Nguyen, Young-Woo Seo, Kyung Hwa Lee, Jae Hyuk Lee, Kyung Keun Kim, Jae-Seoun Hur, Hangun Kim

**Affiliations:** 1 College of Pharmacy and Research Institute of Life and Pharmaceutical Sciences, Sunchon National University, Sunchon, Republic of Korea; 2 Korean Lichen Research Institute, Sunchon National University, Sunchon, Republic of Korea; 3 Faculty of Natural Science and Technology, Tay Nguyen University, Buon Ma Thuot, Vietnam; 4 Korea Basic Science Institute, Gwangju Center, Gwangju, Republic of Korea; 5 Department of Pathology, Chonnam National University Medical School, Gwangju, Republic of Korea; 6 Medical Research Center for Gene Regulation, Chonnam National University Medical School, Gwangju, Republic of Korea

## Abstract

Lichens are symbiotic organisms that produce various secondary metabolites. Here, different lichen extracts were examined to identify secondary metabolites with anti-migratory activity against human lung cancer cells. *Everniastrum vexans* had the most potent inhibitory activity, and atranorin was identified as an active subcomponent of this extract. Atranorin suppressed β-catenin-mediated TOPFLASH activity by inhibiting the nuclear import of β-catenin and downregulating β-catenin/LEF and c-jun/AP-1 downstream target genes such as CD44, cyclin-D1 and c-myc. Atranorin decreased KAI1 C-terminal interacting tetraspanin (KITENIN)-mediated AP-1 activity and the activity of the KITENIN 3′-untranslated region. The nuclear distribution of the AP-1 transcriptional factor, including c-jun and c-fos, was suppressed in atranorin-treated cells, and atranorin inhibited the activity of Rho GTPases including Rac1, Cdc42, and RhoA, whereas it had no effect on epithelial-mesenchymal transition markers. STAT-luciferase activity and nuclear STAT levels were decreased, whereas total STAT levels were moderately reduced. The human cell motility and lung cancer RT² Profiler PCR Arrays identified additional atranorin target genes. Atranorin significantly inhibited tumorigenesis *in vitro* and *in vivo*. Taken together, our results indicated that *E. vexans* and its subcomponent atranorin may inhibit lung cancer cell motility and tumorigenesis by affecting AP-1, Wnt, and STAT signaling and suppressing RhoGTPase activity.

## Introduction

Lung cancer is the leading cause of cancer-related death worldwide, and approximately 85% of cases are related to cigarette smoking^1^. Metastasis, which is common in lung cancer, is a multi-stage process involving invasion into surrounding tissue, intravasation, transit in the blood or lymph, extravasation, and growth at a new site^2^. Many of these steps require cell motility, and increased cell motility such as migration and/or invasion can lead to cancer progression. Adjacent invasion and distant metastasis are the major causes of lung cancer-related death^3^. The aim of the present study was to search for potential inhibitors of migration and invasion to improve the survival of patients with lung cancer.

Lichens are symbiotic organisms that are usually composed of a fungal partner and a photosynthetic partner^4^. Lichen is a known source of approximately 800 unique secondary metabolites, which are produced by the fungus and secreted onto the surface of hyphae either in amorphous form or as crystals^5^. The intense antioxidant activity of lichens plays important ecological roles, and they possess antibiotic, anti-proliferative, and cytotoxic activities. These secondary products are frequently used by the pharmaceutical industry as antibacterial and antiviral compounds^5, 6^. Lichens and their secondary metabolites have been studied for their anticancer properties. However, a limited number of lichen substances have been screened for their biological activities and their therapeutic potential in anticancer medicine^7^. The current study examined five lichen species collected from Vietnam, China, and Chile for their inhibitory activity against the migratory and invasive abilities of human lung cancer cells and investigated the mechanisms underlying the inhibitory activity of lichen substances against lung cancer cell motility and tumorigenesis.

## Results

### Inhibition of A549 cell motility by acetone extracts of lichens

**Figure 1:**
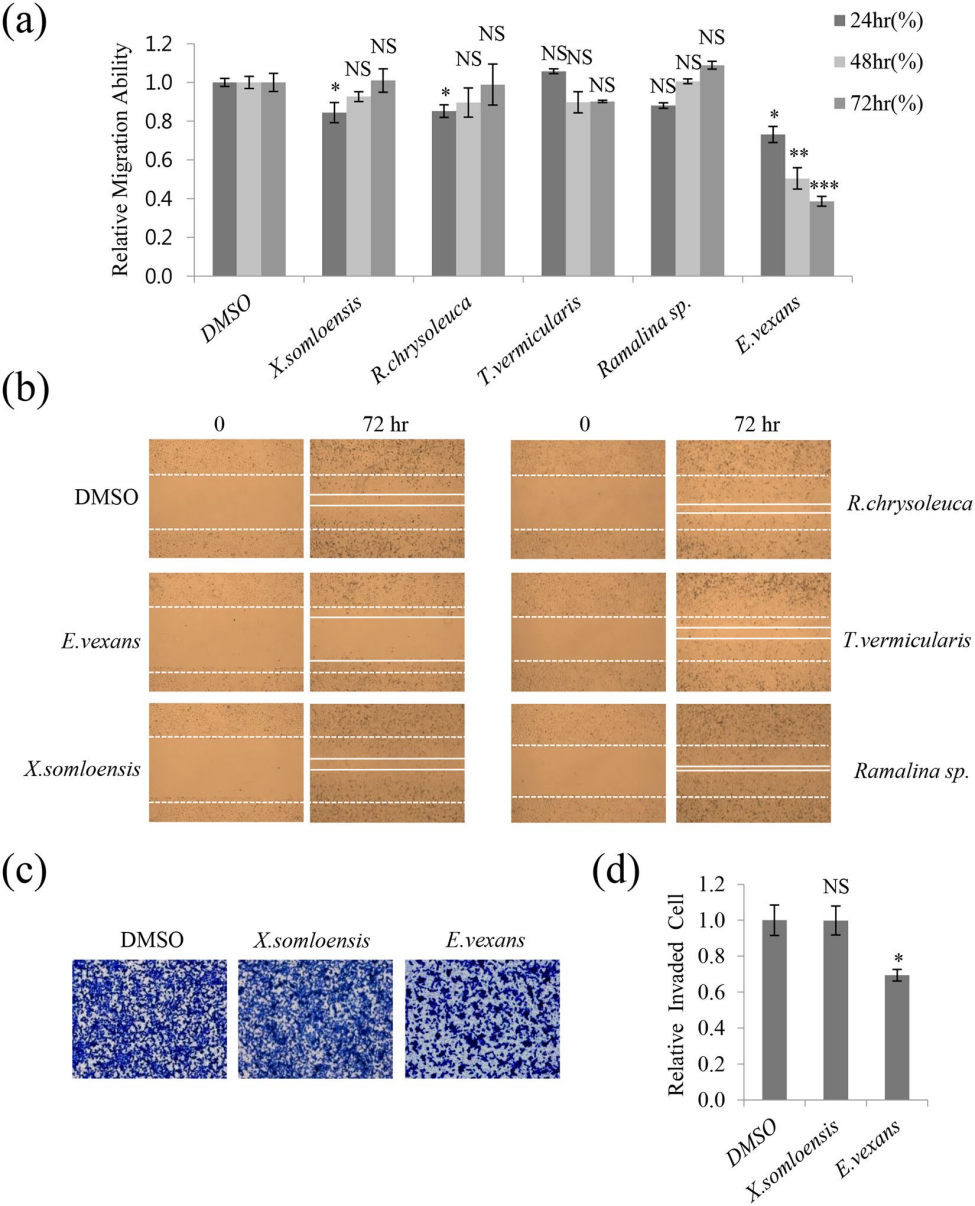
Lichen crude extracts inhibited A549 cell migration and invasion. (**a**,**b**) Quantitative analysis and representative images of migration assays in A549 cells treated with 10 μg/mL acetone extracts of *Xanthoparmelia somloensis*, *Rhizoplaca chrysoleuca*, *Thamnolia vermicularis*, *Ramalina sp*., and *Everniastrum vexans*. (**c**,**d**) Invasion assays in A549 cells treated with 10 μg/mL acetone extracts of *X. somloensis* and *E. vexans*, and quantitative analysis of invaded cell numbers in each group. Representative images from three independent experiments are shown (n = 3). Data represent the mean ± S.E.M. *p < 0.05; **p < 0.01; ***p < 0.001; NS, no significant difference compared with dimethylsulfoxide (DMSO)-treated A549 cells.

Migration and invasion play a crucial role in the metastasis of cancer cells. To identify inhibitory substances among lichen secondary metabolites, acetone extracts of five types of lichens were screened using wound healing assays in A549 human lung cancer cells (Supplementary Table). As shown in Fig. 1a, only *Everniastrum vexans* (VN140298) inhibited the migration of A549 cells at a concentration of 10 μg/mL. This concentration was not cytotoxic and was used for subsequent assays (data not shown). The length between the edges of the wound at 72 h with *E. vexans* (VN140298) was significantly wider than those with DMSO or the non-active samples *Xanthoparmelia somloensis* (CH130062), *Rhizoplaca chrysoleuca* (CH130190), *Thamnolia vermicularis* (CH130219-1), and *Ramalina sp*. (CL130494). In particular, *E. vexans* (VN140298) showed more than 60% inhibitory activity compared with the control (Fig. 1a and b).

To determine whether *E. vexans* (VN140298) had inhibitory activity against invasion in A549 cells, invasion assays were performed using gelatin-coated chambers. The number of invaded cells was approximately 30% lower in samples treated with *E. vexans* than in those treated with DMSO or *X. somloensis* (CH130062) (negative control) (Fig. 1c and d). These findings indicated that acetone extracts of *E. vexans* (VN140298) inhibited the migratory and invasive abilities of A549 lung cancer cells.

### Atranorin was identified as an active secondary metabolite from *E. vexans* with inhibitory activity against A549 cell motility

**Figure 2:**
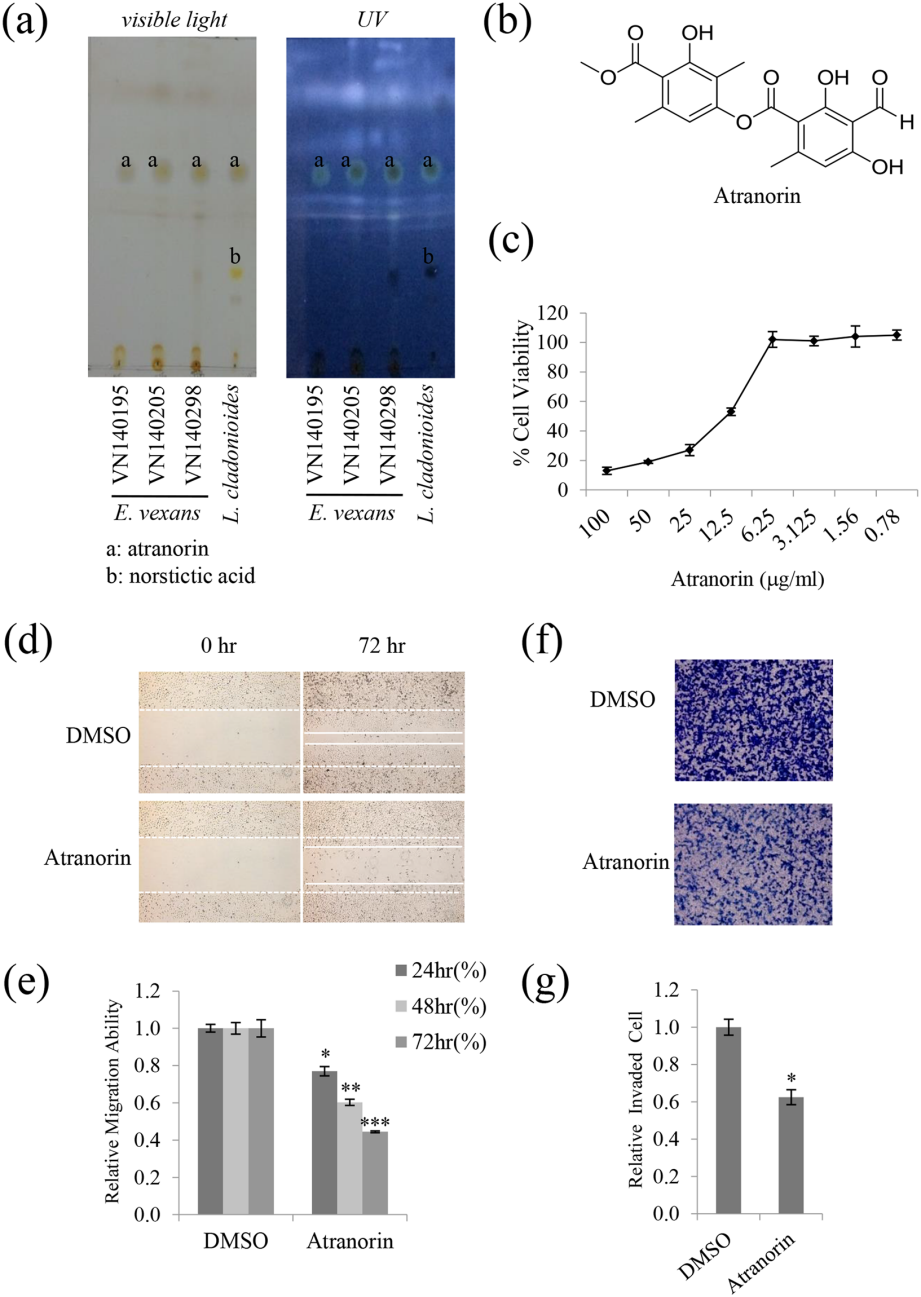
Atranorin was identified as an active secondary metabolite from *E. vexans* with inhibitory activity against A549 cell motility. (**a**) TLC analysis performed using a Toluene: Dioxin: Acetic acid = 180: 45: 5 (v/v/v) solvent system showed that lichen extracts had inhibitory activity against A549 cell motility; ‘a’ denotes the location of the spot for atranorin. *L. cladonioides* was used as the standard control for atranorin; it contained atranorin (spot ‘a’) and norstictic acid (spot ‘b’). (**b**) Chemical structure of atranorin. (**c**) MTT assay in A549 cells treated with atranorin at different doses. (**d**,**e**) Migration assay in A549 cells treated with 5 μg/mL atranorin, and quantitative analysis of wound length. (**f**,**g**) Invasion assays in A549 cells treated with 5 μg/mL atranorin and quantitative analysis of invaded cell numbers in each treatment. Quantitative data were obtained from three independent experiments (n = 3). Data represent the mean ± S.E.M. *p < 0.05; **p < 0.01; ***p < 0.001 compared with DMSO-treated A549 cells.

To identify the subcomponents of the acetone extract of lichens, *E. vexans* (VN140195, VN140205, and VN140298) extracts were individually analyzed by thin layer chromatography (TLC) (Fig. 2a). Based on the Rf values, atranorin was the main compound identified in these candidates after comparison with *Lethariella cladonioides* (Nyl.) Krog (Atranorin). As ‘spot a’ in *E. vexans* (VN140195, VN140205, and VN140298) shared an identical TLC Rf value with atranorin in *L. cladonioides* (Nyl.) Krog and the same position and color under daylight and UV light (left and right panels, Fig. 2a), ‘spot a’ was identified as atranorin^8, 9^. The atranorin used in this study was purchased from ChromaDex (CA, USA), and the chemical structure is shown in Fig. 2b. The cytotoxicity of atranorin was measured in A549 cells by the MTT assay, which showed that atranorin was not cytotoxic at 5 μg/mL, whereas it showed remarkable cytotoxicity at concentrations of >5 μg/mL (Fig. 2c).

To determine whether atranorin was the active compound inhibiting A549 cell motility, wound healing and invasion assays were performed. In accordance with that *E. vexans* inhibited the migratory and invasive abilities of A549 cells (Fig. 1), atranorin at 5 μg/mL inhibited migration by 46% and invasion by 33% (Fig. 2d–g). These results demonstrated that atranorin, a secondary metabolite of *E. vexans*, had potential inhibitory activity against A549 cell motility.

### Atranorin decreased β-catenin-mediated TOPFLASH activity by suppressing β-catenin nuclear import and downregulated β-catenin/LEF and c-jun/AP-1 downstream target genes

**Figure 3:**
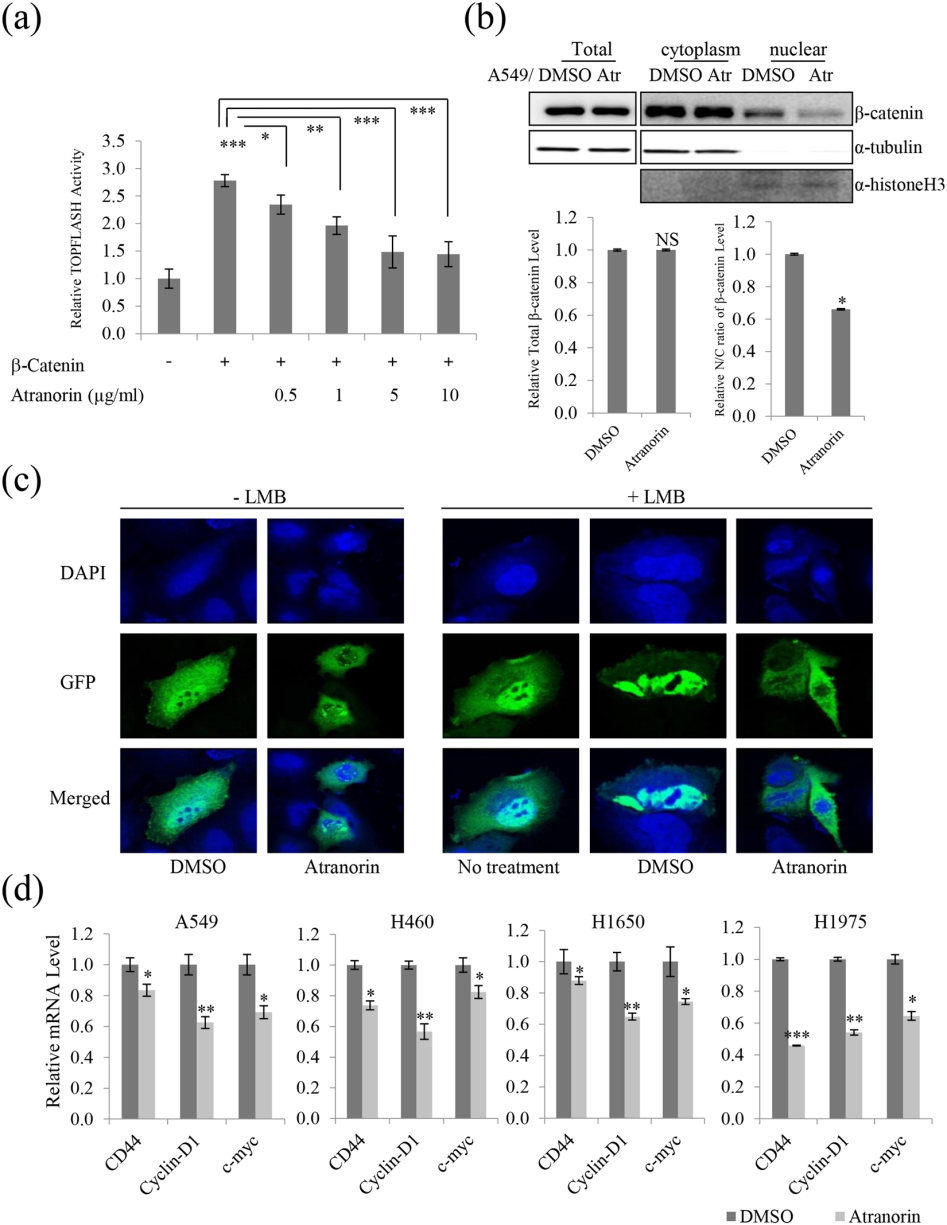
Atranorin inhibited β-catenin-mediated TOPFLASH activity by suppressing nuclear import and downregulated β-catenin/LEF and c-jun/AP-1 downstream genes. (**a**) Atranorin decreased the β-catenin-mediated transcriptional activity of the TOPFLASH promoter. HEK293T cells were transfected with β-catenin and the TOPFLASH reporter plasmid. After 12 h of transfection, cells were treated with atranorin for 48 h. (**b**) Decreased β-catenin nuclear localization upon atranorin treatment. The total, nuclear, and cytoplasmic levels of β-catenin were analyzed in A549 cells. α-Histone H3 was used as a nuclear marker. Quantitative analysis of the ratio of nuclear to cytoplasmic β-catenin in A549 cells treated with 5 μg/mL atranorin. (**c**) A549 cells were transiently transfected with GFP-β-catenin for 12 h. Cells were visualized using a fluorescence confocal microscope after DMSO or atranorin treatment for 24 h (left). Treatment with leptomycin B (LMB, a nuclear export inhibitor) alone induced a significant accumulation of nuclear β-catenin at 4 h (central). Treatment with DMSO resulted in significant retention of nuclear β-catenin for 24 h after 4 h of pretreatment with LMB, whereas the accumulation was not observed after treatment with atranorin (right). DAPI was used for visualization of the nucleus. (**d**) Quantitative analysis of the mRNA level of CD44, c-myc, and cyclin-D1 in A549, H460, H1650, and H1975 cells treated with 5 μg/mL atranorin. Data represent the mean ± S.E.M. (n = 3). *p < 0.05; **p < 0.01; ***p < 0.001 compared with the DMSO-treated group in each cell line.

The Wnt pathway affects multiple biological processes, especially cell motility^10^. Wnt-mediated pathways have been proposed that function independently of β-catenin, which acts as a major downstream effector^11^. Atranorin at concentrations of 0.5–10 μg/mL decreased β-catenin-mediated TOPFLASH activity by 18–50% in a dose-dependent manner (Fig. 3a). Assessment of the nuclear/cytoplasmic distribution of β-catenin showed that the β-catenin nuclear to cytoplasmic ratio was remarkably lower in atranorin- than in DMSO-treated cells, whereas the level of total β-catenin remained unchanged (Fig. 3b). This indicated that atranorin inhibited the nuclear localization of β-catenin. To confirm this, cells were transfected with endogenous GFP-β-catenin to characterize the nuclear localization pattern of β-catenin in response to atranorin treatment for 24 h (Fig. 3c). The results showed that cells treated with atranorin contained reduced nuclear β-catenin compared with those treated with DMSO (left two panels). Treatment with Leptomycin B (LMB), a specific inhibitor of β-catenin nuclear export, for 4 h showed a dramatic accumulation of nuclear β-catenin (+LMB, central panel). In the two DMSO-treated groups, LMB significantly increased nuclear β-catenin compared with non-LMB treated cells, whereas LMB did not increase nuclear β-catenin in the atranorin-treated groups. The analysis of tumor cells in Fig. 3c indicated that atranorin suppressed the nuclear import of β-catenin. Upon translocation to the nucleus, β-catenin interacts with T-cell factor 4 (TCF4) and lymphoid enhancer-binding factor 1 (LEF1) to regulate the expression of a wide range of genes at the transcriptional level^12^. The complex binds to the A-C/G-A/T-T-C-A-A-A-G motif, which is an evolutionarily conserved consensus motif on the promoter of target genes^13^. While c-myc, cyclin-D1, and CD44 expression is known to be regulated by this complex^14^, multiple new targets have recently been identified. To determine whether the levels of downstream target genes of β-catenin/LEF and c-jun/AP-1 were affected by atranorin treatment, qRT-PCR analysis was performed. As shown in Fig. 3d, the relative expression levels of CD44, cyclin-D1, and c-myc were significantly decreased by atranorin treatment in lung cancer cells.

### Atranorin affected the expression of KAI1 and KITENIN and downregulated downstream transcriptional factors

**Figure 4:**
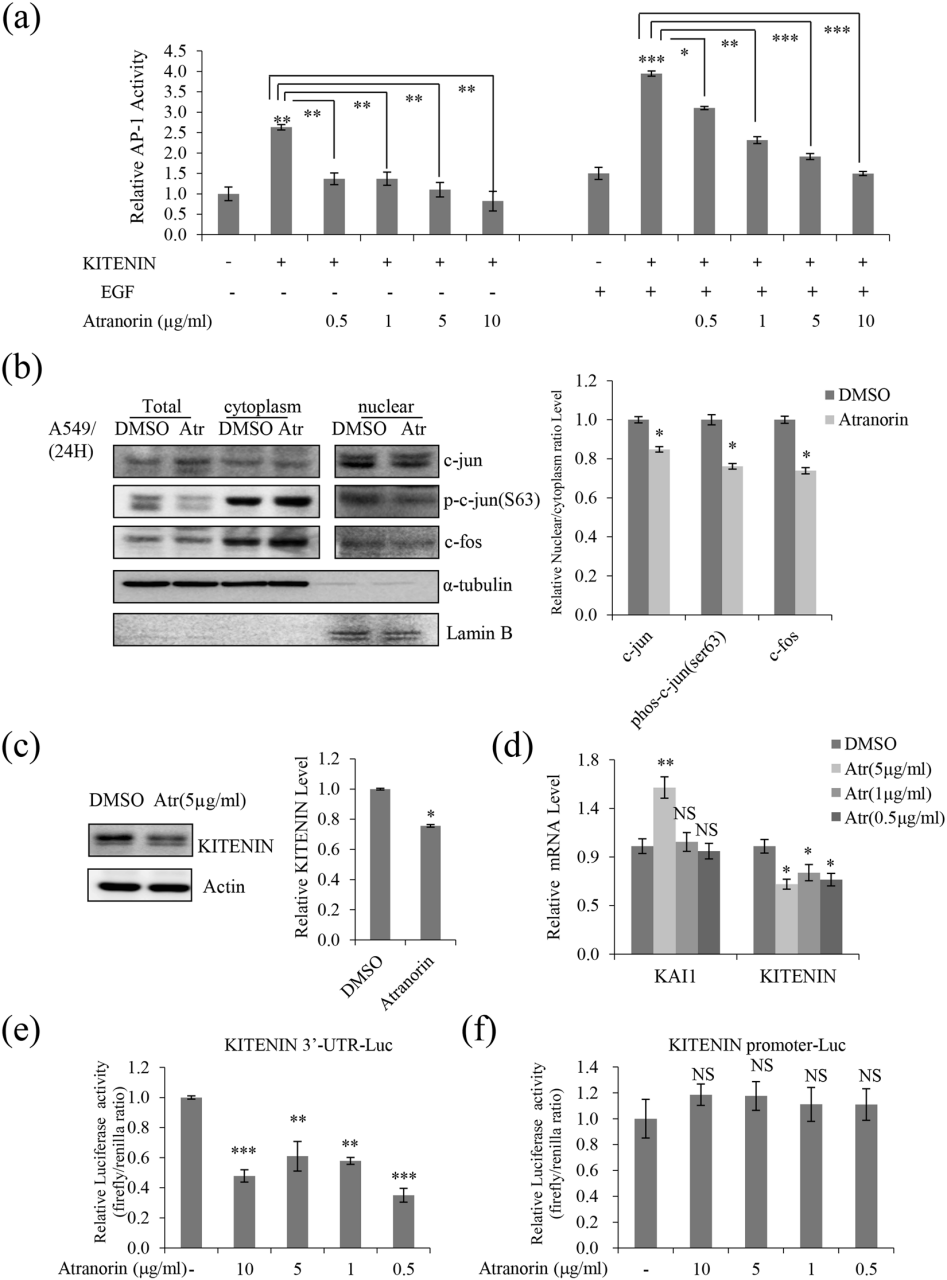
Atranorin suppressed KITENIN-mediated AP-1 activity, affected the expression of KAI1 and KITENIN, and downregulated c-jun and c-fos. (**a**) Atranorin inhibited the KITENIN-mediated transcriptional activity of the AP-1 promoter. After 12 h of transfection with KITENIN and the AP-1 reporter plasmid, cells were treated with atranorin for 48 h in the presence or absence of EGF. (**b**) Western blot analysis of total, cytoplasmic, and nuclear c-jun, phospho-c-jun (ser63), and c-fos in A549 cells. (**c**) Western blot analysis of KITENIN in A549 cells treated with 5 μg/mL atranorin. (**d**) Quantitative analysis of the mRNA level of KITENIN and KAI1 in A549 cells treated with different concentrations of atranorin. (**e,f**) KITENIN 3′-UTR (**e**) and promoter (**f**) luciferase assays in HEK293T cells treated with atranorin. Quantitative data were obtained from at least two independent experiments. Data represent the mean ± S.E.M. (n = 3). *p < 0.05; **p < 0.01; ***p < 0.001; NS, no significant difference compared with the DMSO-treated group in each cell line.

KITENIN promotes the tumorigenic potential of cancers, and epidermal growth factor (EGF) stimulates KITENIN-mediated AP-1 activation^15, 16^. Atranorin decreased AP-1 activity in a dose-dependent manner starting at 0.5 μg/mL, and a significant decrease of approximately 70% was observed in response to 10 μg/mL. EGF-activated KITENIN-mediated AP-1 activity was also decreased by atranorin treatment (Fig. 4a). The activity of the AP-1 transcriptional factors c-jun and c-fos is regulated by phosphorylation at Ser63 or Ser73 through the SAPK/JNK pathway. Examination of the nuclear/cytoplasmic distribution of c-jun and c-fos showed that the nuclear to cytoplasmic ratio of c-fos and c-jun, especially that of phospho-c-jun (Ser63), was lower in DMSO-treated than in atranorin-treated cells at 5 μg/mL (Fig. 4b). KITENIN expression levels were also suppressed, as shown in Fig. 4c. The metastatic suppressor gene, KAI1, is often downregulated in metastatic tumor cells^17, 18^. As KITENIN has an inverse relationship with KAI1, we tested the mRNA level of KAI1 in response to increasing doses of atranorin. The results showed that atranorin suppressed the mRNA expression of KITENIN and significantly upregulated KAI1 mRNA expression (Fig. 4d). Taken together, these results suggested that atranorin inhibited A549 cell motility partly by suppressing KITENIN-mediated AP-1 activity through the modulation of KITENIN and KAI1 expression in lung cancer cells. To further examine the regulation of KITENIN by atranorin, luciferase assays were performed. The results showed that atranorin decreased the activity of the KITENIN 3′-UTR (Fig. 4e), whereas the KITENIN promoter was not affected (Fig. 4f). This suggested that the suppressive effects of atranorin on KITENIN were associated with the activation of KAI1 expression, and atranorin inhibited lung cancer cell motility by modulating KITENIN-mediated signaling.

### Atranorin affected several additional cell motility-related factors in lung cancer cells, including the activity of Rho GTPases, STAT, and the expression of related genes

**Figure 5:**
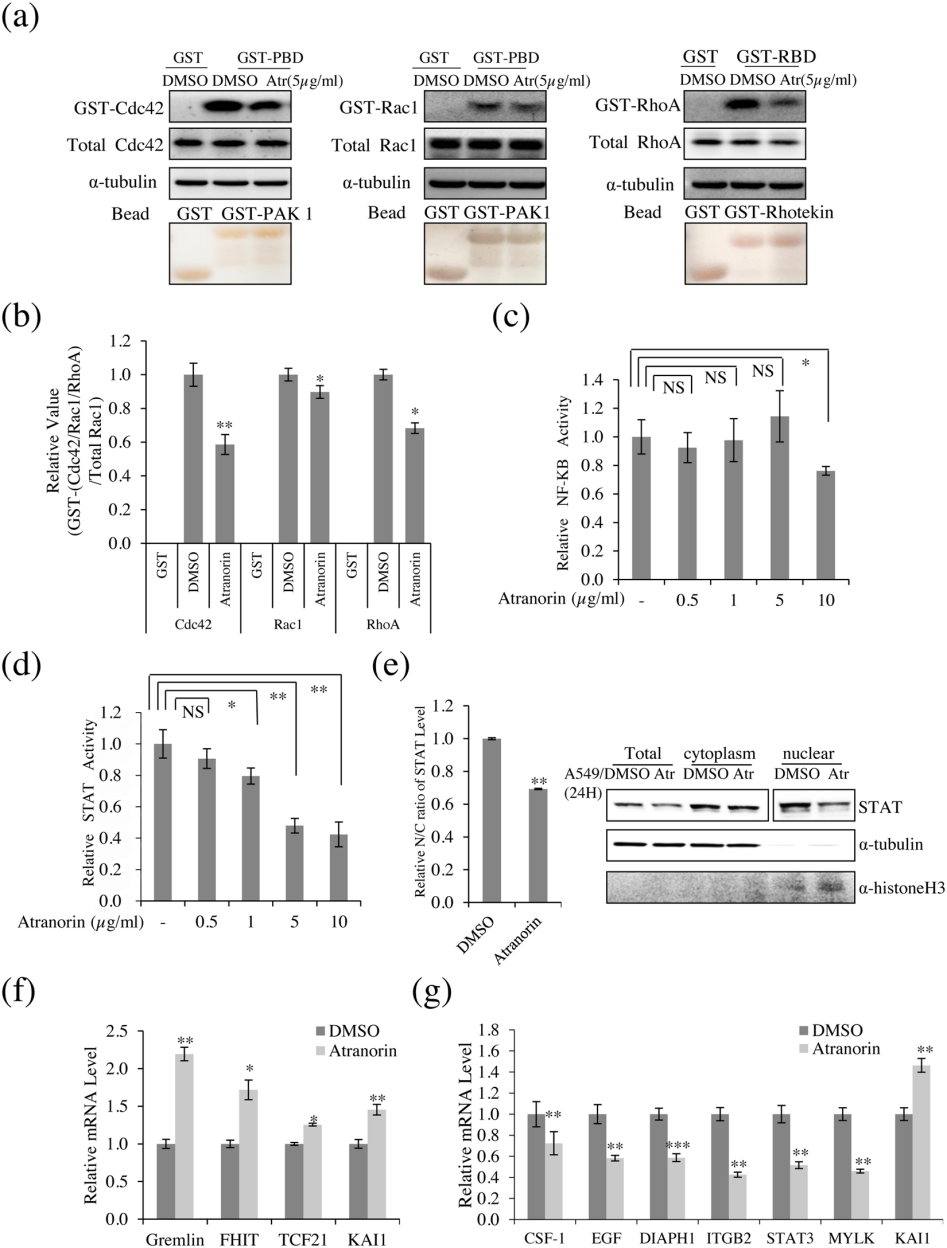
Atranorin reduced RhoGTPase activity and affected the expression of cell motility and lung cancer-related genes. (**a**) The levels of GTP-bound Cdc42, Rac1, and RhoA were measured in A549 cells treated with 5 μg/mL atranorin. GTP-Rac1 and -Cdc42 were measured using GST-PBD, and GTP-RhoA was measured using GST-RBD. The total amounts of RhoA, Rac1, and Cdc42 are also shown. (**b**) The relative activities of Cdc42, Rac1, and RhoA were measured as described in Materials and Methods. (**c**) NF-κB luciferase assay of HEK293T cells treated with 5 μg/mL atranorin. (**d**) STAT-transfected HEK293T cells showed a dose-dependent decrease in luciferase activity. (**e**) Western blot analysis of total, cytoplasmic, and nuclear STAT. (**f**) Quantitative analysis of the mRNA levels of Gremlin, FHIT, TCF21, and KAI1 in A549 cells treated with 5 μg/mL atranorin. (**g**) Quantitative analysis of the mRNA levels of CSF1, EGF, DIAPH1, ITGB2, STAT3, MYLK, and KAI1 in A549 cells treated with 5 μg/mL atranorin. KAI1 levels were measured as a positive control for atranorin activity. The data represent the mean ± S.E.M. (n = 3). *p < 0.05; **p < 0.01; ***p < 0.001; NS, no significant difference compared with DMSO-treated A549/HEK293T cells.

RhoA, Rac1, and Cdc42 are involved in the mesenchymal mode of migration^19, 20^. The effect of atranorin on the activities of these proteins in A549 cells was examined by GST pull-down assays with GST-PBD (p21-binding domain) and GST-RBD (Rho-binding domain). As shown in Fig. 5a and b, atranorin significantly decreased the level of GTP-Cdc42 by 42% compared with that in vehicle-treated cells, and the activity of Rac1 was decreased by 13% in the presence of atranorin. RhoA promotes junction formation and apical constriction, and reduces adhesion and cell spreading^21^. Atranorin treatment significantly decreased the level of GTP-RhoA by 32% compared with that in vehicle-treated cells. Taken together, these results suggested that atranorin inhibited cell motility by regulating Rho GTPases, whereas it had no effect on the levels of epithelial-mesenchymal transition markers (Supplementary Figure). Assessment of NF-κB (NF-κB-luc) and STAT (STAT-luc) in treated cells (Fig. 5c and d) showed that atranorin decreased STAT-luciferase reporter gene activation in a dose-dependent manner (Fig. 5d), whereas atranorin decreased NF-κB activity only at 10 μg/mL (Fig. 5c). In addition, atranorin significantly decreased the nuclear STAT level compared with that in DMSO-treated cells, and moderately reduced the total STAT level (Fig. 5e). Next, the RT² Profiler™ PCR Array was used to examine genes related to lung cancer and lung cancer cell motility, respectively. Three genes related to lung cancer were screened: Gremlin, fragile histidine triad (FHIT), and transcription factor 21 (TCF21). Gremlin is an inhibitor of the TGF-β signaling pathway that is considered a potential therapeutic and diagnostic target in human cancers^22^. FHIT synergizes with VHL, another tumor suppressor, to protect against chemically induced lung cancer^23^. TCF21 is also deregulated in several types of cancers and functions as a tumor suppressor (Fig. 5f). Six positive target genes of cell motility were screened: colony-stimulating factor 1 (CSF1), EGF, diaphanous homolog 1 (DIAPH1), β-2 integrin (ITGB2), Signal transducer and activator of transcription 3 (STAT3) and myosin light chain kinase (MYLK). CSF1 is essential for osteoclastogenesis and mediates osteolysis in metastatic tumors^24^. The release of CSF1 may act in an autocrine manner to enhance proliferation and invasion of tumor cells^25^. EGF is used as a metastatic inducer and induces tumor cell invasion and metastasis and the downregulation of Focal Adhesion Kinase^26^. Depletion of DIAPH1 strongly inhibits lung metastasis; in contrast to control cells, DIAPH1-depleted cells do not form metastases in other organs^27^. ITGB2 is known to be responsible for the motility-promoting action, it is integral cell-surface proteins that participate in cell adhesion and cell-surface mediated signaling^28^. STAT3 activation is associated with various cancers and suggests poor prognosis. It can promote oncogenesis and has anti-apoptotic as well as proliferative effects^29^. MYLK is involved in tumor metastasis, when cytokines stimulate the cell surface receptors in tumors, and its mRNA expression in cancer patients with recurrence and distant metastasis was higher than those in the early stage of cancer^30^. Our data indicated that the expression of these cell motility-related genes was downregulated by atranorin in A549 cells (Fig. 5g). Taken together, these results suggested that atranorin is a potential inhibitor of lung cancer cell motility.

### Atranorin inhibited lung cancer invasion and tumor growth *in vitro* and *in vivo*

**Figure 6:**
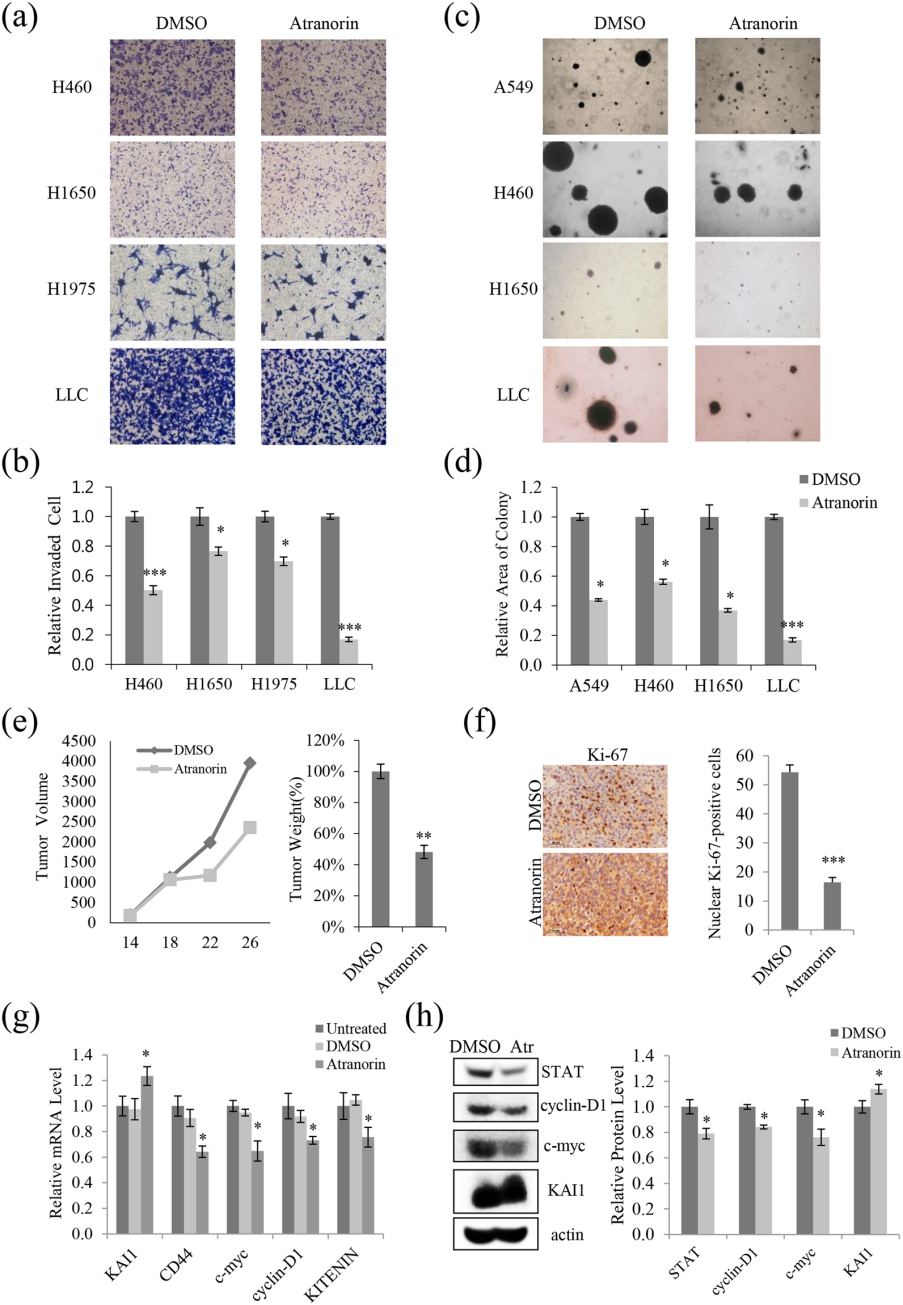
Atranorin inhibited invasion and suppressed tumor growth *in vitro* and *in vivo* in different lung cancer cell lines. (**a**,**b**) Invasion assays in H460, H1650, H1975, and LLC cells treated with 5 μg/mL atranorin, and quantitative analysis of invaded cell numbers in each cell line. (**c**,**d**) Soft agar colony-formation assays in A549, H460, H1650, and LLC cells treated with atranorin (5 μg/mL) and quantitative analysis of colony areas in each group. (**e**) LLC cells were inoculated subcutaneously into C57BL/6 mice (n = 5 per group) at 2 × 10^6^ cells per mouse. On day 12 of treatment, tumor volume at different time points and tumor weight were measured (p < 0.005). (**f**) Immunohistochemistry of tumor tissue sections using a Ki-67 antibody displayed nuclear Ki-67 immunoreactivity, a marker of cell proliferation. (**g**,**h**) Mouse tissue lysates were analyzed for the expression of KAI1, CD44, c-myc, cyclin-D1, and KITENIN by qRT-PCR (**g**) or for the expression of STAT, cyclin-D1, c-myc, and KAI by Western blot (**h**). Representative images are shown from three independent experiments (n = 3). Data represent the mean ± S.E.M. *p < 0.05; **p < 0.01; ***p < 0.001 compared with untreated or DMSO-treated cells.

The inhibitory activity of atranorin in additional lung cancer cell lines was examined by performing invasion assays in H460, H1650, H1975, and LLC cells. Atranorin treatment significantly decreased the number of invaded H460, H1650, H1975, and LLC cells (Fig. 6a). Quantitative analysis showed that atranorin inhibited invasion by 50%, 24%, 30%, and 80% in H460, H1650, H1975, and LLC cells, respectively, compared with vehicle-treated cells (Fig. 6b). A soft agar colony-formation assay was performed to test whether sub-lethal concentrations of atranorin inhibited anchorage-independent growth of lung cancer cells. As shown in Fig. 6c and d, colony formation from A549, H460, H1650, and LLC cells on soft agar was significantly decreased by treatment with atranorin. Results from an *in vivo* xenograft model further confirmed that atranorin reduced tumor volume, weight, and Ki-67 immunoreactivity (Fig. 6e and f). Consistent with previous results, the main target genes, such as KITENIN, STAT, c-myc, CD44, and/or cyclin-D1 were suppressed *in vivo* (Fig. 6g and h). Taken together, these results demonstrated that atranorin has antitumorigenic activity in lung cancer cells.

## Discussion

Lichens are unique organisms that produce biologically active metabolites with a variety of effects. In the present study, we investigated the inhibitory effect of lichen substances on the migration and invasion of lung cancer cells. Of five lichen species analyzed, *E. vexans* had the most potent inhibitory activity against A549 cell motility. TLC analysis identified atranorin as the main active compound of this lichen. Atranorin is the main compound and an important member of the depside group, which is found in a variety of lichen species^31^. It was identified as 3-hydroxy-4-methoxycarbonyl-2,5-dimethylphenyl-3-formyl-2,4-dihydroxy-6-methylbenzoate^32^. Atranorin has been studied extensively as a lichen metabolite with potential anticancer properties, including anti-inflammatory^33^, apoptotic cell death^34^, and cell cycle arrest^35^ effects; however, its biological activity against lung cancer motility has not been reported to date. The findings of the present study are as follows: 1) The acetone extract of *E. vexans* inhibited lung cancer cell motility, and the subcomponent, atranorin, showed similar inhibitory activity against migration and invasion of lung cancer cells. 2) Atranorin decreased β-catenin-mediated TOPFLASH activity and altered subcellular β-catenin distribution by suppressing its nuclear import. Atranorin downregulated the downstream target genes of β-catenin/LEF and c-jun/AP-1. 3) Atranorin decreased KITENIN-mediated AP-1 activity and suppressed the expression of the downstream factors c-jun and c-fos. 4) Atranorin reduced RhoGTPase and STAT activity, and affected target genes associated with metastatic potential and involved in lung cancer development. 5) Atranorin inhibited tumorigenesis *in vitro* and *in vivo*. We demonstrated for the first time that atranorin has inhibitory activity against lung cancer cell motility and tumorigenesis and elucidated the underlying mechanism of action.

The Wnt pathway controls tissue polarity and cell movement^36^. The control of β-catenin levels is a key feature of the canonical Wnt/β-catenin signaling pathway. Upon Wnt pathway activation, unphosphorylated β-catenin accumulates in the cytoplasm and translocates into the nucleus, where it interacts with TCF and LEF to activate the transcription of Wnt/β-catenin target genes^37^. The target genes include cell cycle-regulating genes (c-myc and cyclin-D1) and genes related to metastasis and the invasion of cancer cells^38^. Our reporter assays showed that atranorin effectively regulated β-catenin by suppressing its nuclear import, and inhibited the Wnt/β-catenin signaling pathway stimulated by β-catenin/TCF interaction, suggesting that atranorin might target β-catenin or its downstream effectors (Fig. 3).

KAI1 is a metastasis suppressor gene^39^, and KITENIN is a newly identified binding partner of the KAI1/CD82 metastasis suppressor^40^. KITENIN promotes tumorigenesis, invasiveness, and adhesion to fibronectin in mouse colon cells^41^. The present results showed that atranorin decreased KITENIN-mediated AP-1 activity and downregulated KITENIN expression, inhibiting the 3′-UTR activity of KITENIN (Fig. 4). Recently, it was reported that EGF increases KITENIN-induced AP-1 activity, and c-Jun and c-fos belong to the dimeric AP-1 family of transcription factors, which are critical regulators of gene expression^42^. The KITENIN/ErbB4-Dvl2-c-Jun axis is an unconventional EGFR-independent downstream signal of EGF and mediates the invasiveness and tumorigenesis of cancer cells^15, 43^. However, our results showed that atranorin decreased phospho-c-jun and c-fos nuclear distribution and inhibited the anchorage-independent growth of lung cancer cells. This suggests that atranorin may have potential beneficial activity in overcoming the limited clinical efficacy of anti-EGFR therapy.

Rho GTPases contribute to multiple cellular processes that affect cancer progression, including proliferation, survival, invasion, and metastasis^44, 45^. The members of the Ras superfamily of small GTPases, such as Rac1, Cdc42, and RhoA, are adhesion and growth factor-activated molecular switches that play important roles in tumor development and progression^46^. RhoA, Rac 1, and Cdc42 also play a role in cell-cell adhesion in epithelial cells in addition to their effects on the actin cytoskeleton and motility^46^. Our results showed that the activities of Rho GTPases were decreased by atranorin, suggesting that atranorin plays an important role in the inhibition of cell motility. In the canonical JAK-STAT pathway, JAK phosphorylates and activates STAT, leading to the nuclear translocation of phospho-STAT^47^. In the present study, atranorin suppressed nuclear STAT activity and the mRNA level of STAT3, as shown by RT² Profiler PCR Array. We used the human Cell Motility RT² Profiler PCR Array, involved in the movement of cells, and the Human Lung Cancer RT² Profiler PCR Array, involved in lung cancer development, as screening resources for cancer gene discovery. Our results showed that atranorin regulated the expression of CSF-1, EGF, DIAPH1, ITGB2, STAT3, MYLK, gremlin, FHIT, and TCF21. These genes are associated with metastatic potential, and genes differentially expressed in lung cancer may provide insight into the molecular mechanisms underlying lung oncogenesis (Fig. 5). The present findings identified some of the molecular mechanisms underlying the anticancer activity of atranorin, such as Wnt signaling, KITENIN-mediated AP-1 activity, Rho GTPase regulation, and STAT activity.

Atranorin significantly suppressed tumorigenic potential in a mose xenograft tumor model and reduced nuclear Ki-67 level which is a prototypic cell cycle related nuclear protein expressed in proliferating cells in all phase of the active cell cycle. Also, molecular mechanisms of anticancer activity for atranorin presented in this study were confirmed *in vivo*. Together with recent literature showing anticancer activity of atranorin^48, 49^, our findings provide insight into the anticancer activity of lichen species; further study is required to determine the potential clinical application on lung cancer therapy.

## Methods

### Preparation of lichen extracts

Lichen specimens were collected from Vietnam/China/Chile in 2014 and identified at the Korean Lichen Research Institute (KoLRI)^50^. The dried thalli of the lichens were extracted with acetone at room temperature for 48 h and then filtered and dried in a rotary vacuum evaporator at 45 °C. The dry extracts were dissolved in dimethylsulfoxide (DMSO) for all experiments.

### Thin layer chromatography (TLC) analysis

Lichen thalli were soaked in acetone, and the concentrated solution was spotted. Solvent A, described in Culberson’s improved standardized method^51^, was used in this study. The results were visualized by examination and marking in daylight (for pigments) and under UV light at 254 and 350 nm. The lichen species *Lethariella cladonioides* (Nyl.) Krog (Atranorin) was used as the standard control. Based on the standardized method, the relative Rf value was determined to help identify each spot.

### Cell culture

The lung cancer cell lines A549, H1650, H1975, H460, and Lewis lung carcinoma (LLC) were obtained from the Korean Cell Line Bank (Seoul, South Korea). Cells used in the study were authenticated by a commercial service (Korean Cell Line Bank) with short tandem repeat profiling. Cells were cultured in RPMI 1640/DMEM culture medium (Gen Depot, TX, USA) supplemented with 10% fetal bovine serum (Gen Depot, TX, USA) and 1% Penicillin-Streptomycin solution. Cells were cultured in 5% CO_2_ in a humidified atmosphere at 37 °C.

### MTT assay

Cells (2 × 10^4^ cells/well) were seeded on a 96-well plate, grown overnight, and then treated with the acetone extracts and atranorin (ChromaDex, CA, USA) at concentrations of 100 g/mL to 0.78 g/mL for 48 h. After incubation with MTT at 37 °C, cells were lysed with DMSO (Sigma-Aldrich, St. Louis, USA) and absorbance was measured at 570 nm.

### Wound healing assay

A549 cells were plated at a density of 2.5 × 10^5^ cells/well and grown overnight to confluence. Monolayer cells were scratched to create a wound. The cells were then washed twice and incubated in RPMI1640 culture medium supplemented with 2% FBS with 10 μg/mL of the lichen extract or 5 μg/mL atranorin. For the quantitation of relative migration ability, photographs of cells were taken at 0, 24, 48, and 72 h after wounding to measure the width of the wound. The distance migrated by the cells was calculated as the difference between the edges of the wound at time point 1 and at time point 2. For each sample, an average of five wound distances was taken to determine the average rate of migration at a given concentration of lichen extract or atranorin. The migrating rate was determined with the following formula: migrating rate [%] = (width t_1_ [mm] - width t_2_ [mm]) / width t_1_ × 100%.

### Invasion assay

Invasion assays were performed in Transwell chambers (Corning, New York, USA) with an 8 µm pore polycarbonate membrane coated with 1% gelatin. Invasion assays were performed as previously described^16^. For the quantitation of relative invaded ability, stain the cells adhering to the under-side of the filter and count the number of cells in different five fields of view to get an average sum of cells. Invaded rate [%] = (stained area-sample [mm²] / stained area-control [mm²]) × 100%. Each invasion assay was repeated in three independent experiments. The results are expressed as the mean number of cells migrating per high-power field.

### Soft agar colony-formation assay

Cells (1 × 10^4^) were suspended in soft agar (0.35% agarose), plated onto solidified agar (0.6% agarose) in 6-well plates, and cultured for 3 weeks. Cells were treated twice per week with atranorin (5 g/mL) and DMSO (0.01%). Pixel intensity of the colony area was measured with IMT i-Solution software (IMT i-Solution Inc., Northampton, NJ, USA). To calculate the colony area percentage, the diameter of each colony was quantified as colony size. Data represent the mean of three experiments.

### Reporter assay

The Renilla luciferase^®^ reporter assay was performed as previously described^16^. The KAI1 C-terminal interacting tetraspanin (KITENIN) promoter (−2344 to +536) was amplified by PCR from genomic DNA of HEK293T cells using primers designed from the genomic sequence of the KITENIN locus. The KITENIN 3′-untranslated region (3′-UTR) assay was performed as previously described^52^. Briefly, a 2.3-kb DNA fragment of the KITENIN 3′-UTR was cloned into the pMIR-REPORT luciferase vector (Ambion, Austin, TX) downstream of the reporter gene. Fold changes were calculated using values normalized to Renilla luciferase activity.

### Western blotting

Cells were treated with 5 μg/mL atranorin for 24 h and lysed, and the extracted protein (25 μg) was separated by 8% SDS-PAGE. Bands were measured by Multi-Gauge 3.0, and their relative density was calculated based on the density of the α-tubulin or actin bands in each sample. Values were expressed as arbitrary densitometric units corresponding to signal intensity.

### Cell fractionation

Cytoplasmic and nuclear extracts were separated with the NE-PER nuclear and cytoplasmic extraction kit (PIERCE Biotechnology, USA). Cells were harvested and resuspended in ice-cold CER I, and then ice-cold CER II was added. Lysates were homogenized and centrifuged at 16000 × g for 5 min at 4 °C, and supernatants were used as cytoplasmic fractions. The remaining lysates were resuspended in ice-cold NER for 40 min, and the soluble fraction was centrifuged at maximum speed; the supernatant (nuclear extract) was collected.

### qRT-PCR

Briefly, total RNA was isolated from human lung cancer cells using RNAiso Plus (TaKaRa, Otsu, Shiga 520–2193, Japan) according to the manufacturer’s instructions. Total RNA (1 μg) from each group of treated cells was converted to cDNA using a M-MLV Reverse Transcriptase Kit (Invitrogen, Carlsbad, USA) and SYBR green (Enzynomics, Seoul, Korea). qRT-PCR reaction and analysis were performed using CFX (Bio-Rad, Hercules, USA).

### Affinity precipitation of cellular GTPases

Cellular RhoA, Rac1, and Cdc42 activities were determined using GST-RBD/PBD as previously described^53^. The relative activity of each GTPase was determined by quantifying each band of GTP-bound GTPase and the total amount of GTPase, and the values of the GTP-bound bands were normalized to the value of the total amount.

### RT² Profiler™ PCR array

The human Cell Motility RT² Profiler™ PCR Array was used to profile the expression of 84 key genes related to cell motility according to the manufacturer’s instructions. The Human Lung Cancer RT² Profiler PCR Array was used to profile the expression of 84 key genes commonly involved in lung cancer development. The fold change of mRNA expression was calculated on the basis of the cycle threshold^13^ values obtained from the RT-PCR experiment. The scatter plot of test versus control samples indicated the validity of the experiment.

### Xenograft mouse model

The experimental protocol was approved by the Sunchon National University Research Institutional Animal Care & Use Committee. Maintenance of animals and all *in vivo* experiments were performed according to the Guiding Principles in the Care and Use of Animals (DHEW publication, NIH 80–23). LLC cells (2 × 10^6^ cells) were subcutaneously injected into the flanks of 8-week-old C57BL/6 mice. Drug treatment was initiated 14 days after tumor cell injection, at which time the primary tumor had reached approximately 50 mm^3^. Animals were treated with 40% DMSO in PBS (vehicle) or 20% atranorin (10 mg/kg) mixed with 20% DMSO every 3 days by intraperitoneal injection for 2 weeks. Tumors were measured every 3 days with an electronic caliper. When primary tumors reached approximately 300 mm^3^, tumors were surgically removed for further analysis. The tumor volume was determined with the following formula: tumor volume [mm^3^] = (largest diameter [mm]) × (smallest diameter [mm])² × 0.52. The tumors were histologically examined, and the tissue sections were deparaffinized; rehydrated; rinsed; hybridized with Ki-67 antibodies; and examined as described^54^.

### Statistical analysis

All experiments were performed multiple times. Data were expressed as the mean ± standard error of the mean. All statistical analyses were performed using the SPSS version 17. Treatment effects were determined using one-way ANOVA post-hoc analysis. A p-value < 0.05 was considered significant unless indicated otherwise.

## Electronic supplementary material

Dataset 1
