# Author Correction: The lichen secondary metabolite atranorin suppresses lung cancer cell motility and tumorigenesis

**DOI:** 10.1038/s41598-021-91474-y

**Published:** 2021-06-17

**Authors:** Rui Zhou, Yi Yang, So-Yeon Park, Thanh Thi Nguyen, Young-Woo Seo, Kyung Hwa Lee, Jae Hyuk Lee, Kyung Keun Kim, Jae-Seoun Hur, Hangun Kim

**Affiliations:** 1 College of Pharmacy and Research Institute of Life and Pharmaceutical Sciences, Sunchon National University, Sunchon, Republic of Korea; 2 Korean Lichen Research Institute, Sunchon National University, Sunchon, Republic of Korea; 3 Faculty of Natural Science and Technology, Tay Nguyen University, Buon Ma Thuot, Vietnam; 4 Korea Basic Science Institute, Gwangju Center, Gwangju, Republic of Korea; 5 Department of Pathology, Chonnam National University Medical School, Gwangju, Republic of Korea; 6 Medical Research Center for Gene Regulation, Chonnam National University Medical School, Gwangju, Republic of Korea

Correction to: *Scientific Reports* 10.1038/s41598-017-08225-1, published online 15 August 2017

The original version of this Article contained an error in Figure 2D, where the image for Atranorin 0 hr was inadvertently duplicated for the DMSO 0 hr panel.

The original Figure 2 appears below.

**Figure 2:**
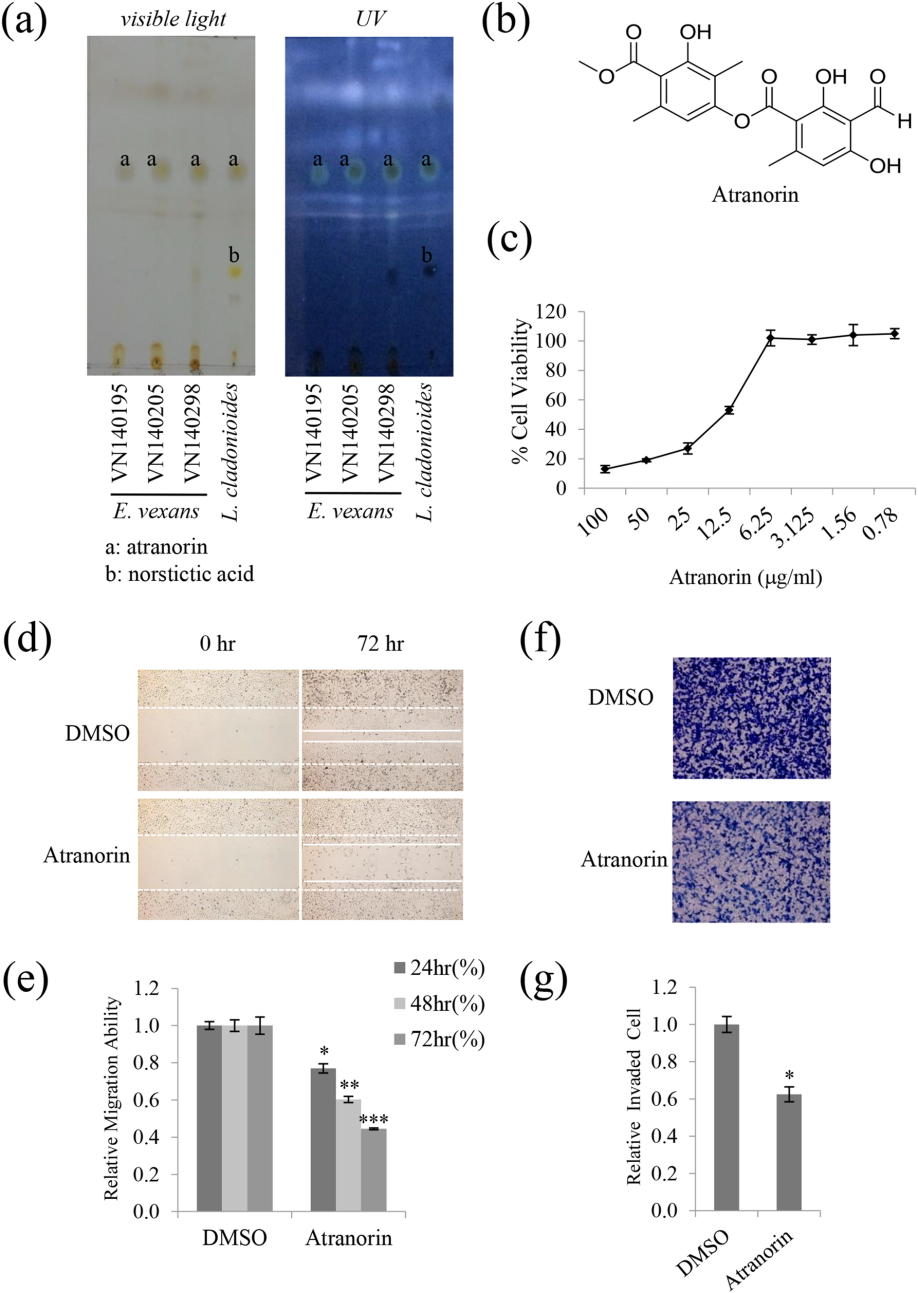
Atranorin was identified as an active secondary metabolite from *E. vexans* with inhibitory activity against A549 cell motility. (**a**) TLC analysis performed using a Toluene: Dioxin: Acetic acid = 180: 45: 5 (v/v/v) solvent system showed that lichen extracts had inhibitory activity against A549 cell motility; ‘a’ denotes the location of the spot for atranorin. *L. cladonioides* was used as the standard control for atranorin; it contained atranorin (spot ‘a’) and norstictic acid (spot ‘b’). (**b**) Chemical structure of atranorin. (**c**) MTT assay in A549 cells treated with atranorin at different doses. (**d**, **e**) Migration assay in A549 cells treated with 5 μg/mL atranorin, and quantitative analysis of wound length. (**f**, **g**) Invasion assays in A549 cells treated with 5 μg/mL atranorin and quantitative analysis of invaded cell numbers in each treatment. Quantitative data were obtained from three independent experiments (n = 3). Data represent the mean ± S.E.M. *p < 0.05; **p < 0.01; ***p < 0.001 compared with DMSO-treated A549 cells.

The original Article has been corrected.

