# Supplementary material for: The lichen secondary metabolite atranorin suppresses lung cancer cell motility and tumorigenesis
Source: Sci Rep. 2017 Aug 15;7:8136. doi: 10.1038/s41598-017-08225-1 (PMC5557893; doi:10.1038/s41598-017-08225-1)

The lichen secondary metabolite atranorin suppresses lung cancer cell motility and tumorigenesis

**Rui Zhou^1^, Yi Yang^1, 2^, So-Yeon Park^1^, Thanh Thi Nguyen^2, 3^, Young-Woo Seo^4^, Kyung Hwa Lee^5^, Jae Hyuk Lee^5^, Kyung Keun Kim^6^, Jae-Seoun Hur^2^, Hangun Kim^1^,***

^1^College of Pharmacy and Research Institute of Life and Pharmaceutical Sciences, Sunchon National University, Sunchon, Republic of Korea; ^2^Korean Lichen Research Institute, Sunchon National University, Sunchon, Republic of Korea; ^3^Faculty of Natural Science and Technology, Tay Nguyen University, Buon Ma Thuot, Vietnam; ^4^Korea Basic Science Institute, Gwangju Center, Gwangju, Republic of Korea; ^5^Department of Pathology, Chonnam National University Medical School, Gwangju, Republic of Korea; ^6^Medical Research Center for Gene Regulation, Chonnam National University Medical School, Gwangju, Republic of Korea.

**Supplementary Data**

**Supplementary Table. Seven lichen species from China/Chile/Vietnam used in this study.**

**Supplementary Figure 1. Atranorin did not affect the levels of epithelial-mesenchymal transition markers.** (A-B) Western blot analysis of E-cadherin (A) and N-cadherin (B) in A549 cells treated with atranorin. Quantitative analysis of the protein levels of E-cadherin and N-cadherin in A549 cells treated with atranorin were shown in each panel. Quantitative data were obtained from at least two independent experiments. Data represent the mean ± S.E.M. (n = 3). NS, no significant difference compared with the DMSO-treated group in each cell line.

**Supplementary Figure 2. Full-length blots shown in Figure 3.**

**Supplementary Figure 3. Full-length blots shown in Figure 4.**

**Supplementary Figure 4. Full-length blots shown in Figure 5.**

**Supplementary Figure 4. Full-length blots shown in Figure 6.**


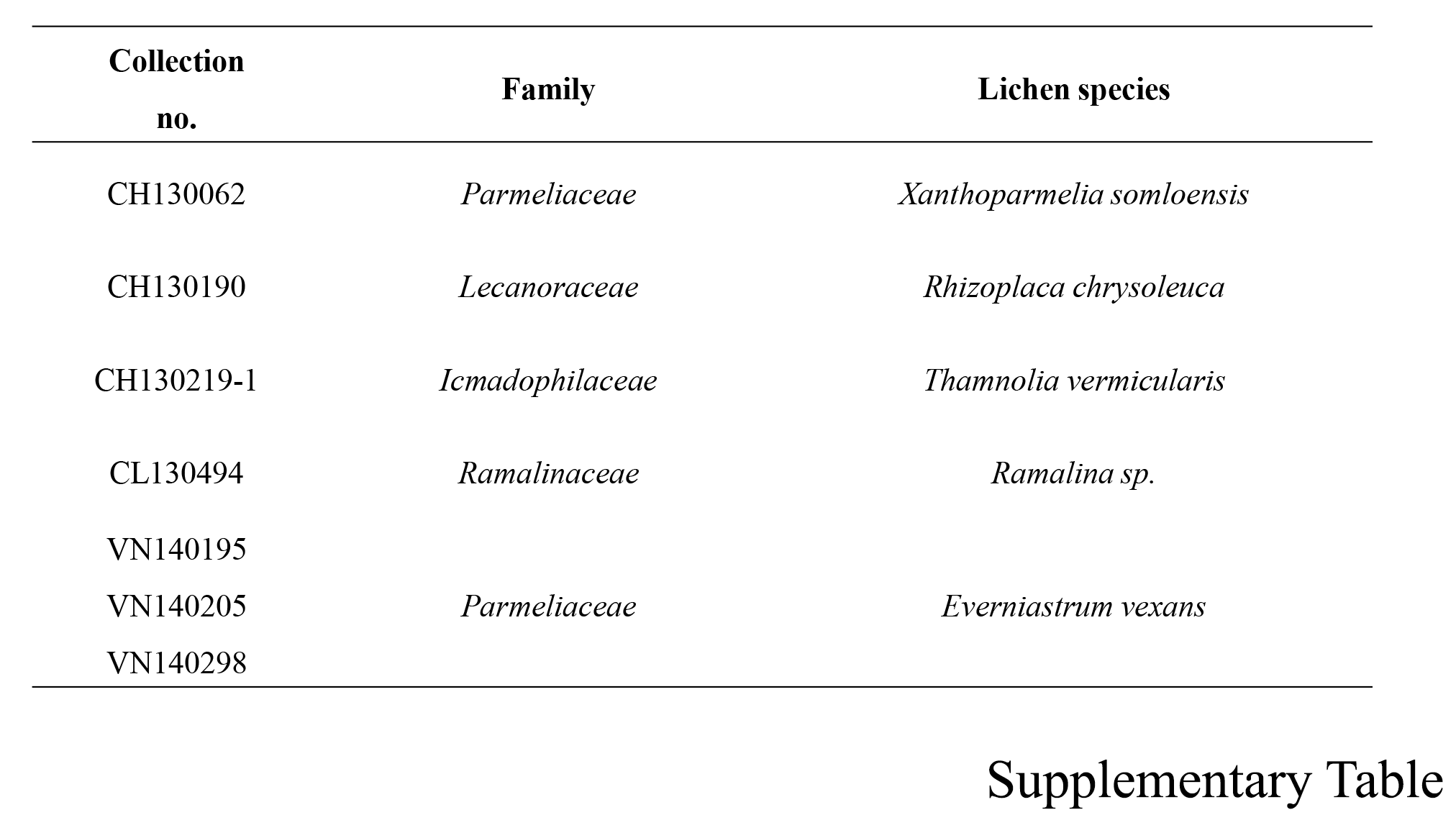


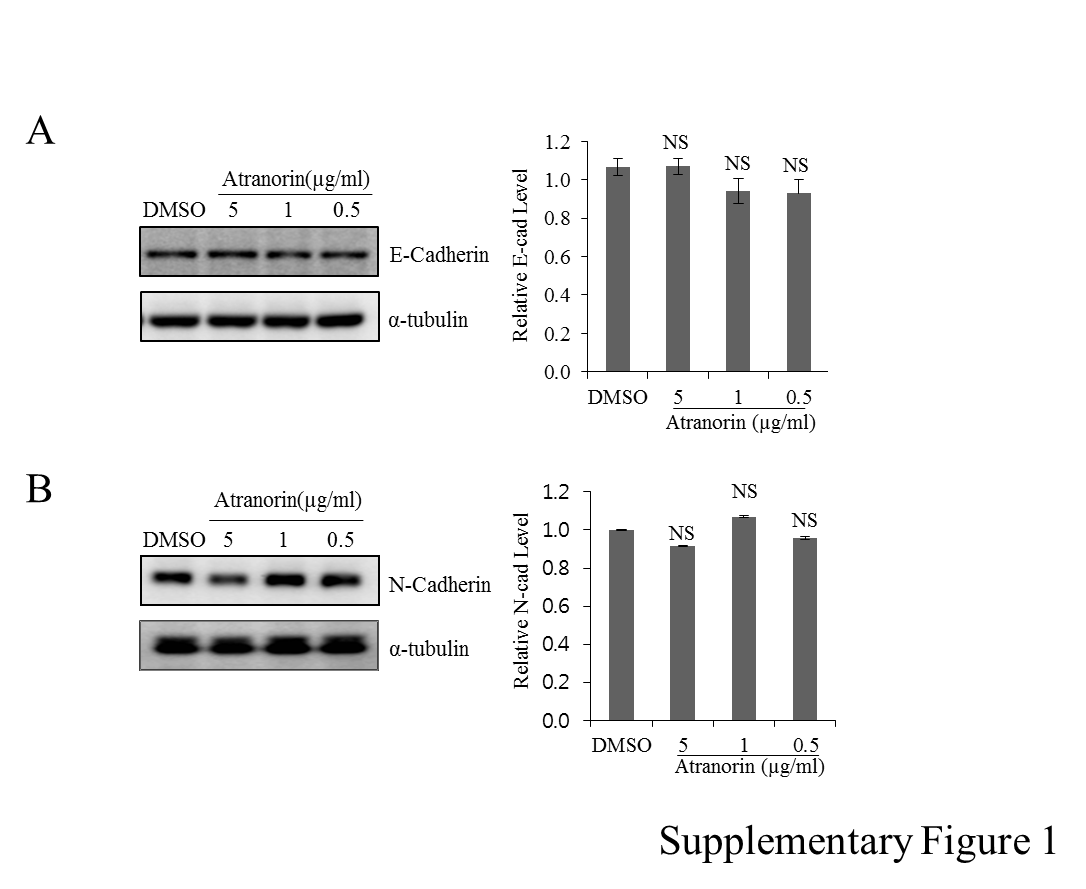


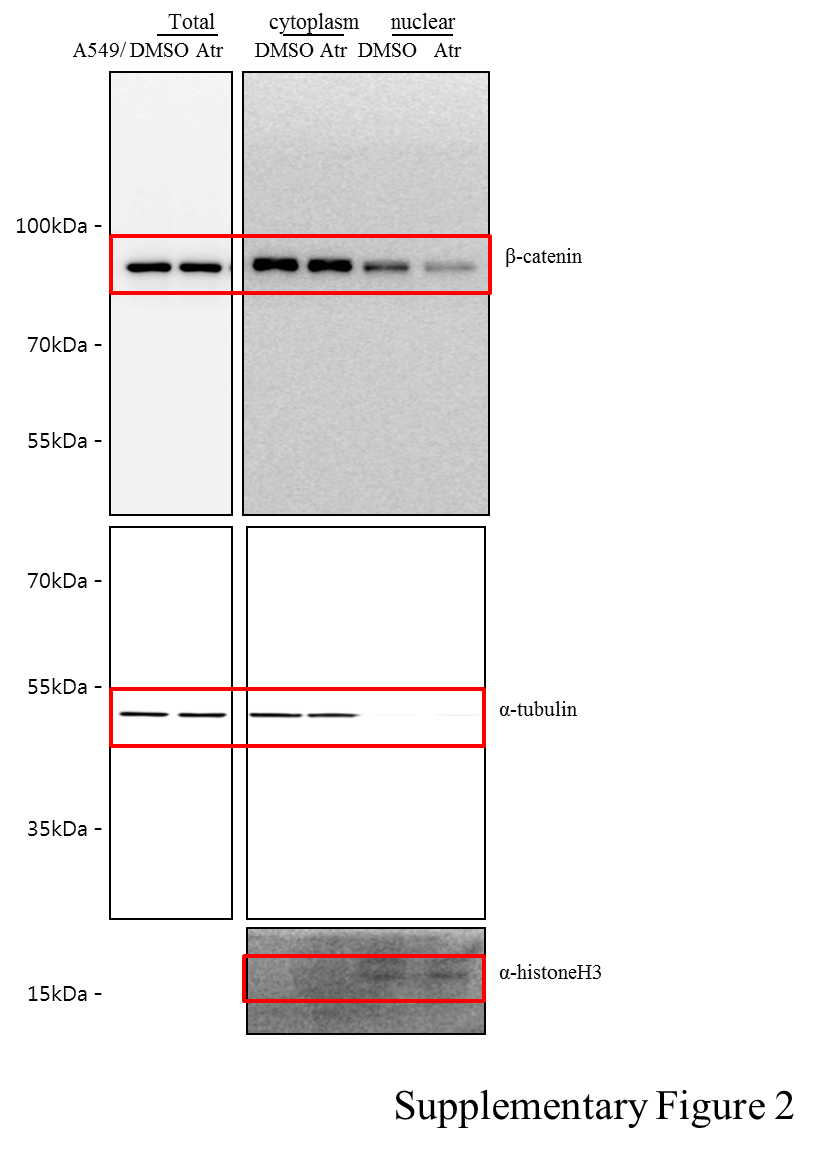


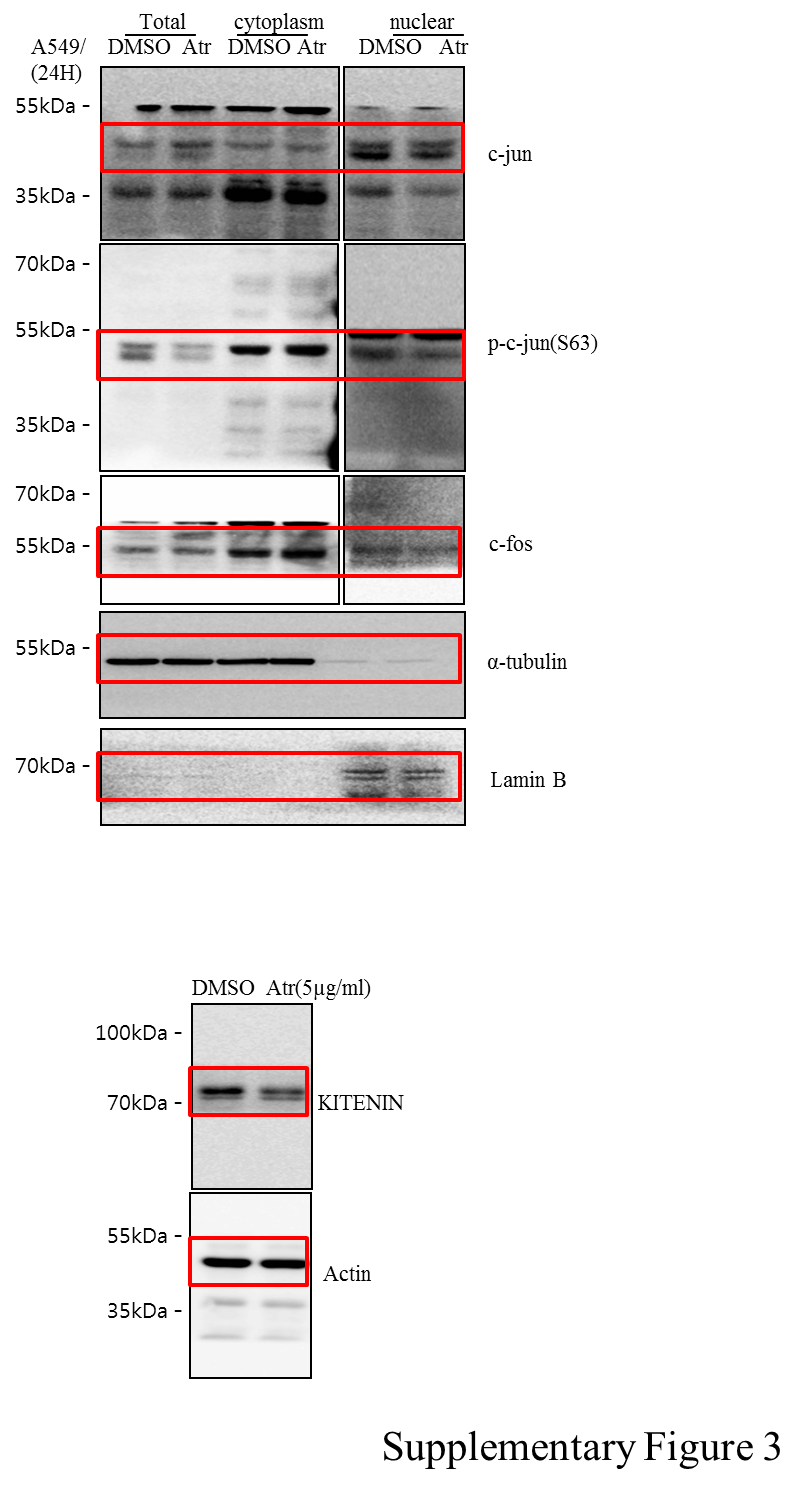


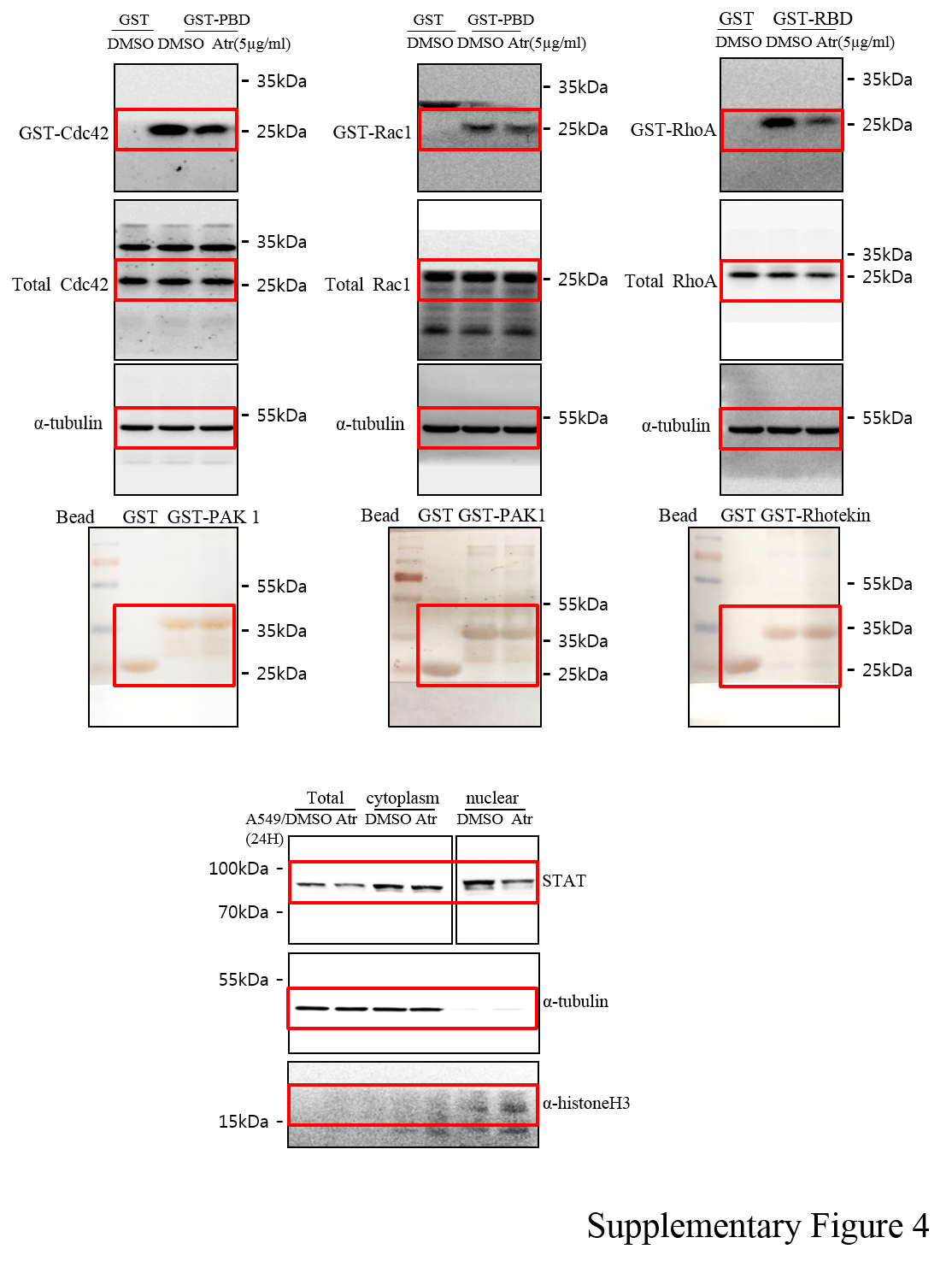


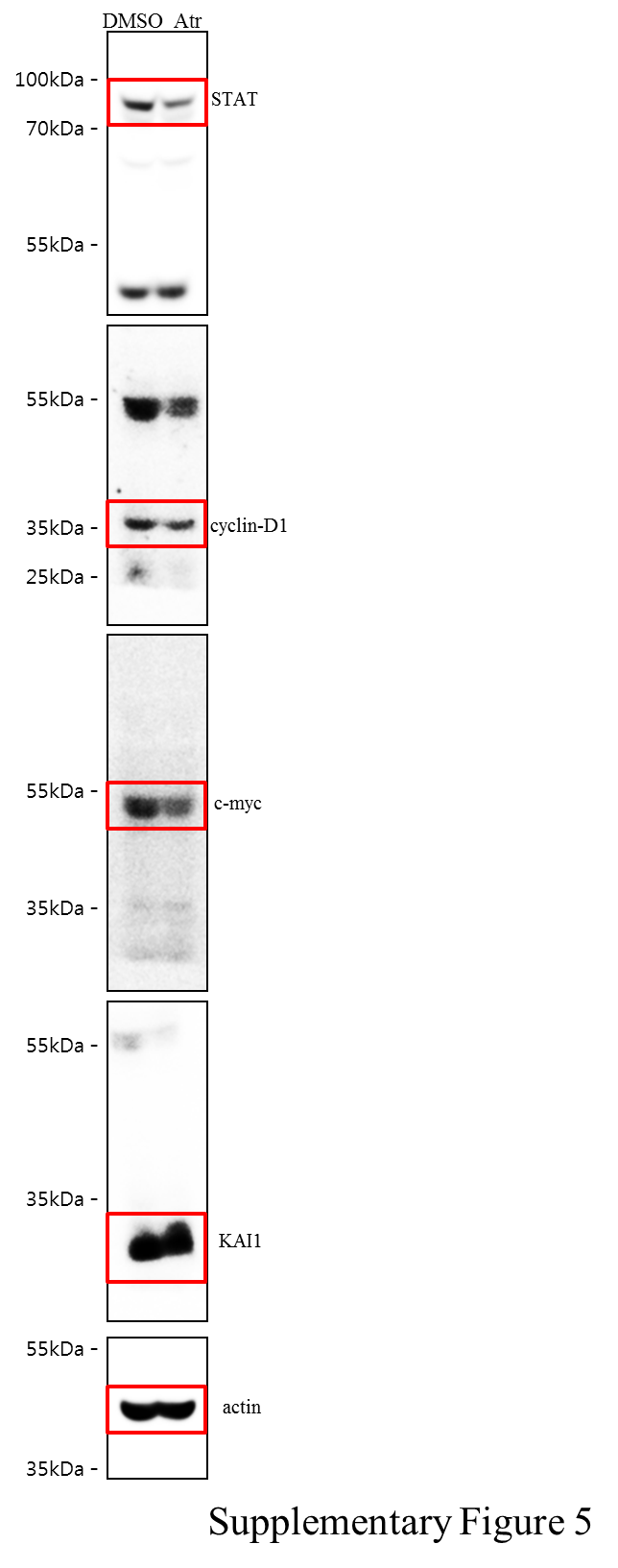

Supplement: Supplementary file 1 — Dataset 1 [file 41598_2017_8225_MOESM1_ESM.doc]
